# Analysing lithium, quetiapine and valproic acid on social media: an infodemiology study

**DOI:** 10.1186/s40345-025-00395-6

**Published:** 2025-10-24

**Authors:** Juan Pablo Chart-Pascual, Miguel Angel Alvarez-Mon, Maria Montero-Torres, Francisco J. Lara-Abelenda, Julen Marin-Napal, Roberto Rodriguez-Jimenez, Raquel Martínez-Velasco, Iñigo Alberdi-Paramo, Ana Gonzalez-Pinto, Cesar I. Fernandez-Lazaro

**Affiliations:** 1 Department of Child and Adolescent Psychiatry, Institute of Psychiatry, Psychology and Neuroscience, Kings College London, London, UK; 2 Psychiatry Department, Osakidetza Basque Health Service, Araba University Hospital, Vitoria-Gasteiz, Spain; 3 University of the Basque Country UPV/EHU, Vitoria-Gasteiz, Spain; 4 Bioaraba Research Institute, Vitoria-Gasteiz, Spain; 5 CIBERSAM, Madrid, Spain; 6 Department of Medicine and Medical Specialities, University of Alcalá, Alcalá de Henares, 28801 Madrid, Spain; 7 Department of Psychiatry and Mental Health, Hospital Universitario Puerta de Hierro, 28222 Madrid, Spain; 8 Ramón y Cajal Institute of Sanitary Research (IRYCIS), 28034 Madrid, Spain; 9 Department of Signal Theory and Communications and Telematic Systems and Computing, School of Telecommunications Engineering, Rey Juan Carlos University, Madrid, Spain; 10 Instituto de Psiquiatría y Salud Mental, Hospital Clínico San Carlos de Madrid, Madrid, Spain; 11 Departamento de Psiquiatría y Medicina Legal, Facultad de Medicina, Universidad Complutense de Madrid, , Madrid, Spain; 12 Research Institute Hospital, 12 de Octubre (imas12), Madrid, Spain; 13 Department of Legal Medicine, Psychiatry, and Pathology, Complutense University of Madrid, Madrid, Spain; 14 Department of Preventive Medicine and Public Health, School of Medicine, University of Navarra, 31008 Pamplona, Spain; 15 IdiSNA, Navarra Institute for Health Research, 31008 Pamplona, Spain

## Abstract

**Background:**

Although lithium is considered the gold standard for the maintenance treatment of bipolar disorder (BD), its prescription has declined in recent decades. At the same time, second-generation antipsychotics (SGAs), such as quetiapine, and other mood stabilisers such as valproic acid, have been increasingly used. Social media platforms such as X (formerly Twitter) provide real-time insights into public and professional perceptions of these treatments, which may influence their use and adherence.

**Aims:**

To analyse how lithium, quetiapine, and valproic acid have been represented on X, by focusing on user type, engagement levels, and thematic content of tweets.

**Method:**

We conducted a mixed-methods, observational study of tweets published in English and Spanish between 2008 and 2022. Tweets containing the generic or commercial names of lithium, valproic acid, and quetiapine were retrieved and analysed using a validated codebook and semi-supervised machine-learning models. Tweets were categorised by user type and clinical and non-clinical content themes.

**Results:**

Among the 236,797 analysed tweets, quetiapine was the most frequently mentioned drug (69.4%), followed by valproic acid (19.1%) and lithium (11.5%). Lithium tweets showed the highest engagement (54.0 likes and 18.0 retweets per tweet). Patients mainly focused on quetiapine (47.0%), while healthcare professionals more often discussed lithium (58.1%). Tweets containing clinical content were more common in English (78.0%) than in Spanish (54.7%), especially regarding side effects (53.1% vs 8.2%). Tweets on effectiveness were more frequently discussed in English (48.8%), especially for quetiapine (54.7%), but were less common in Spanish (9.8%). Discussion about drug shortages was more prevalent in Spanish tweets (31.6% vs 0.5%), particularly for valproic acid (55.8%).

**Conclusions:**

Despite lithium being the least mentioned drug, it generated the highest level of engagement, particularly among healthcare professionals. In contrast, quetiapine was widely mentioned by patients, which reflects a more socially widespread and, at times, problematic use. These findings highlight the value of listening to conversations on social media to better understand perceptions, concerns, and attitudes that may influence adherence and prescribing trends in mental health.

**Supplementary Information:**

The online version contains supplementary material available at 10.1186/s40345-025-00395-6.

## Background

Bipolar disorder (BD) is a chronic and debilitating psychiatric condition that affects approximately 0.6% to 1.2% of the global population, typically beginning in late adolescence or early adulthood (Vieta et al. 2018; Merikangas et al. 2011) The condition is characterised by recurrent episodes of mania and depression, requiring long-term pharmacological treatment to prevent relapse, reduce the risk of suicide, and maintain functional capacity (Yatham et al. 2018; Carvalho et al. 2020; Gonzalez-Pinto et al. 2006; Pascual et al. 2023). Current clinical guidelines consistently recommend lithium and second-generation antipsychotics (SGAs), such as quetiapine, as first-line options for maintenance treatment in BD (Yatham et al. 2018; Pascual et al. 2023; Malhi et al. 2015). Among these, lithium is particularly effective, supported by strong evidence of its anti-suicidal properties and its role in preventing the recurrence of mood episodes (Gonzalez-Pinto et al. 2006; Malhi et al. 2015; Cipriani et al. 2013; González-Pinto et al. 2018; González-Pinto et al. 2021).

Despite this evidence, prescription rates for lithium have declined over the past two decades in many countries, including the United States and parts of Europe (Post 2018; Karanti et al. 2016; Pérez de Mendiola et al. 2021). Conversely, there has been a notable rise in the use of SGAs and other mood stabilisers, such as quetiapine or valproic acid (Hayes et al. 2011; Singh et al. 2023). While quetiapine is often employed in bipolar depression and valproic acid in acute mania, their long-term effectiveness may not match that of lithium, particularly in suicide prevention (Gonzalez-Pinto et al. 2006; Malhi et al. 2015; Severus et al. 2014). The reasons for lithium’s underuse include the necessity for regular monitoring of renal and thyroid function, concerns regarding toxicity, and insufficient training among clinicians (Pérez de Mendiola et al. 2021; Malhi et al. 2017; Zorrilla et al. 2023; Alphen et al. 2021). This shift is concerning, given lithium’s status as the gold standard in BD maintenance therapy (Yatham et al. 2018).

Understanding how these medications are perceived by patients, caregivers, and professionals is essential, especially given the persistently low treatment adherence not only in BD but also in other psychiatric conditions where these drugs are commonly used. For instance, quetiapine is also prescribed for schizophrenia and major depressive disorder, as well as for anxiety disorders or insomnia (Taylor et al. 2014). Studies indicate that only about 40% of patients consistently adhere to their pharmacological regimen (Coldham et al. 2002; García et al. 2016), with beliefs and attitudes being more predictive of adherence than pharmacological profiles alone (Scott and Pope 2002).

Traditional research methods, such as surveys, interviews, cohort studies, and naturalistic approaches, have been commonly used to investigate patients' and healthcare providers' experiences with pharmacological treatments for BD, providing important information (Pérez de Mendiola et al. 2021; Singh et al. 2023; Hidalgo-Mazzei et al. 2023). However, these methods have several limitations, including susceptibility to social desirability bias, recall bias, or the inability to gather real-time information (Song et al. 2023; Edwards 2012; Szarfman et al. 2004).

In recent years, social media platforms such as X (formerly Twitter) have emerged as valuable sources of real-time, spontaneous data on public and professional attitudes toward mental health treatments. Several studies have studied using social media content, different public perceptions towards pharmacological and non-pharmacological treatments commonly used in mental health, such as antidepressants, stimulants, psychotherapies or electroconvulsive therapy (Chart-Pascual et al. 2025; Anta et al. 2023; Mon et al. 2021; Mon et al. 2022). Public perceptions towards mental health practitioners have been additionally studied using X, (Battle et al. 2025). showing how social media content analysis offers a unique opportunity to capture authentic, unfiltered discourse, allowing researchers to explore how the general public knows medications and different treatments.

In previous studies conducted by our research group, we have identified the main themes and sentiments expressed in tweets related to psychotropic medications used in BD, including second-generation antipsychotics, lithium, and mood-stabilising anticonvulsants (Chart-Pascual et al. 2024). However, we did not examine in depth the specific content of the tweets, the engagement, or the type of user publishing the clinical and non-clinical content of the discourse. Building on this prior work, the study aims to conduct a detailed content analysis of tweets referring to lithium, valproic acid, and quetiapine to understand better their digital visibility, the type of user behind these tweets, patterns of engagement, and the evolving narratives regarding medical and not medical content that may influence perceptions and clinical use of these medications.

## Methodology

### X data collection strategy

We conducted an observational, mixed-methods investigation to examine the representation of lithium, quetiapine, and valproic acid on the social media platform X. These three medications were selected according to the CANMAT guidelines, which consistently recommend lithium, quetiapine, and valproic acid as first-line options for the treatment of all phases of BD, including acute mania, bipolar depression, and maintenance (Yatham et al. 2018). Lamotrigine, although an important first-line treatment option particularly for bipolar depression, was not included because it is not indicated for the treatment of mania. Furthermore, we also chose these three medications because, in our previous research on social media discourse (Chart-Pascual et al. 2024), they were the drugs that received the highest volume of tweets within their respective pharmacological groups: quetiapine among second-generation antipsychotics, lithium among mood stabilisers, and valproic acid among anticonvulsants (with the exception of lamotrigine). Data were collected using Twitter Binder. This research tool allows comprehensive access to all publicly available tweets. We systematically retrieved tweets published between January 1, 2008, and December 31, 2022, in English and Spanish languages, which contained the generic or commercial names of any of the three medications approved by the regulatory bodies such as the U.S. Food and Drug Administration (FDA) and the European Medicines Agency (EMA). English and Spanish were selected because they are among the most widely used languages on X (Poblete et al. 2011; Alshaabi et al. 2021), providing access to large and diverse datasets across multiple regions. In addition, these are the native or fluent languages of the research team, ensuring accurate manual annotation, interpretation, and validation of the tweets. The full list of keywords used is in Section "Background" of the supplementary material.

### Content analysis process

To analyse the tweet content, we developed a detailed codebook using both deductive and inductive approaches. Deductive categories were drawn from previous studies conducted by our research team (Anta et al. 2023; Carabot et al. 2023), while inductive categories emerged from an exploratory manual analysis of 500 tweets. This initial sample was coded independently by three researchers (JC, JM, RM), who then met to discuss discrepancies and reach a consensus. A fourth researcher reviewed and validated the final version of the codebook (MAAM). Using this validated framework, two researchers each manually classified 3,000 tweets based on content and user type (JM, RM). The manual classification process started by identifying classifiable or unclassifiable tweets. Classifiable tweets were those that were clearly understandable, written in English or Spanish, and related to neuropsychiatric content. Tweets that were vague, lacked meaningful content, or were unrelated to psychiatric topics were considered unclassifiable and excluded from the main analysis.

For all classifiable tweets, we applied two analytical dimensions: user type and content type. *User type* was categorised as one of the following: patient, patient’s acquaintance, healthcare professional, healthcare institution or academic entity, or undetermined. *Tweet content* was grouped into two broad categories: *clinical content* and *other type of content*. *Clinical content* referred to tweets that discussed perceived *drug efficacy*, including whether the medication had a positive or negative effect; *treatment adherence*, such as whether users reported consistently taking the drug or discontinuing it; *inappropriate use*, which included non-therapeutic or recreational mentions of the drug; and *side effects,* describing actual or anticipated adverse reactions. Other content included tweets focused on *economic or legal aspects* related to the medication, such as pricing or regulatory issues; *scientific advocacy or dissemination*, referring to the promotion of evidence-based information; personal inquiries, where users expressed doubts, worries, or sought treatment advice; *trivialisation*, including jokes, sarcasm, or mocking comments about the drug*; drug shortages*, when users reported problems accessing the medication; and tweets that did not fit into any specific content category, labelled as undetermined.

### Machine learning classifier

To extend the manual analysis to the full dataset, we applied a semi-supervised machine-learning approach. Before model training, the tweets underwent preprocessing, including text normalisation, removal of special characters, handling contractions, and elimination of repeated letters or symbols. The manually coded dataset was divided into two subsets: 80% for training the model and 20% for testing. For English-language tweets, we used BERTweet, a transformer-based model specifically pre-trained on over 860 million English tweets. For Spanish-language tweets, we used BETO, a BERT-based model trained on a large corpus of Spanish texts. Both models were fine-tuned using the annotated dataset to replicate the classification made by human experts. Model performance was assessed using the F1 score, which exceeded 0.7 across all categories, indicating robust agreement with human classification. Once validated, both models were deployed to categorise the remainder of the dataset automatically. The complete classification workflow is illustrated in Supplementary S2.

### Statistical analysis

For the statistical analysis, we conducted descriptive analyses to summarise the number and proportion of tweets by drug, user type, content type, and language. Results were visualised using bubble plots to represent tweet volume by user and drug and heat maps to show the distribution of content themes across medications. All analyses were performed using STATA version 15 (StataCorp LP, College Station, TX, USA).

## Results

### Total count of tweets

A total of 236,797 original tweets were included in the analysis, of which 162,032 (68.4%) were tweeted in English and 74,765 (31.6%) in Spanish. Across the overall tweets, quetiapine was the most frequently mentioned drug, accounting for 164,402 tweets (69.4%), followed by valproic acid with 45,198 tweets (19.1%), and lithium with 27,197 tweets (11.5%). Differences between English and Spanish tweets can be observed in Table 1**.** Although lithium was the least frequently mentioned drug, it showed the highest engagement ratios, particularly in Spanish tweets, with a mean of 123.8 likes per tweet and 47.0 retweets per tweet. In contrast, quetiapine—despite being the most mentioned—had lower interaction metrics, especially in English, with only 1.6 likes per tweet and 0.22 retweets per tweet.

**Table 1:** Number of tweets, ratio like/tweet and retweet/tweet discussing lithium, quetiapine, and valproic acid

Drug	Total original tweets (English + Spanish)			English original tweets			Spanish original tweets		
	n (%)	Ratio like:tweet	Ratio retweet:tweet	n (frequency)	Ratio like:tweet	Ratio retweet:tweet	n (frequency)	Ratio like:tweet	Ratio retweet:tweet
Valproic Acid	45,198 (19.1)	0.9	1.2	32,948 (20.3)	0.9	0.2	12,250 (16.4)	1.00	3.3
Lithium	27,197 (11.5)	54.0	18.0	18,996 (11.7)	23.8	5.4	8,201 (11.0)	123.8	80.7
Quetiapine	164,402 (69.4)	1.8	1.1	110,088 (67.9)	1.6	0.2	54,314 (72.7)	2.3	15.9
Total	236,797 (100)			162,032 (100)			74,765 (100)		

### Type of user analysis

**Fig. 1:**
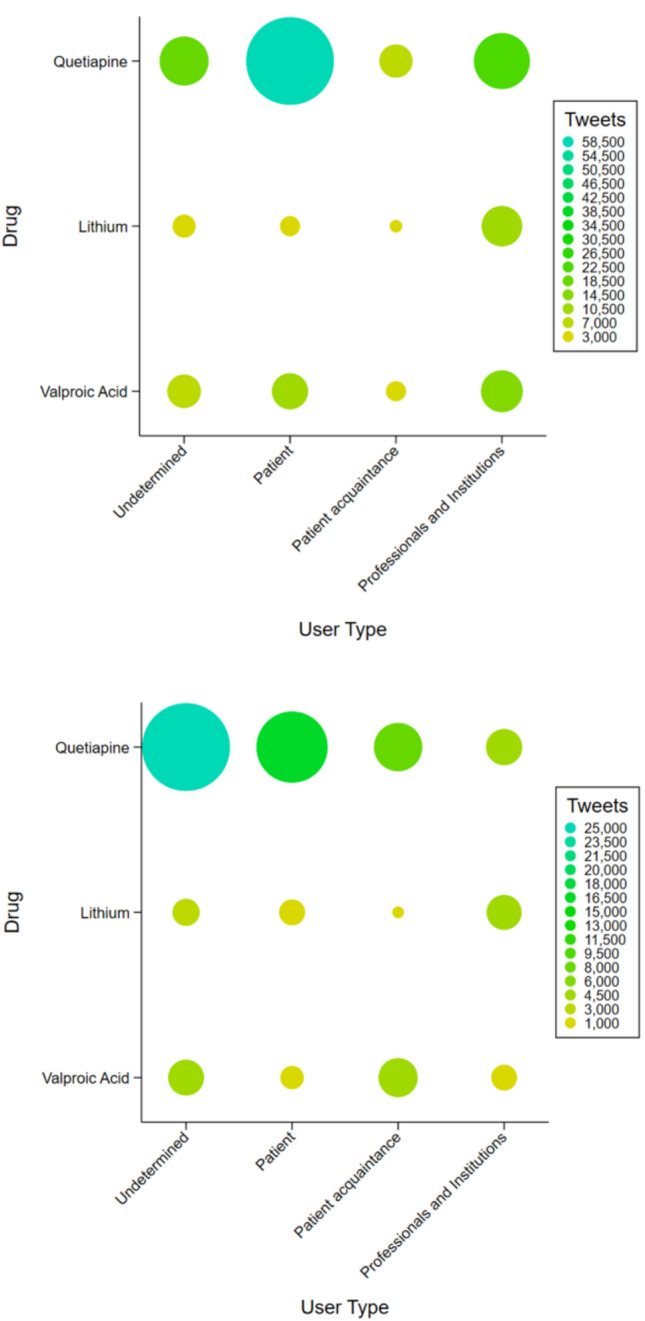
User type analysis posting tweets about lithium, quetiapine and valproic acid. Top panel English tweets. Bottom panel Spanish Tweets

As shown in Fig. 1 quetiapine was the drug most frequently discussed by patients in both languages (54.8% of tweets in English and 31.0% of tweets in Spanish). In contrast, lithium was most commonly tweeted by healthcare professionals, particularly in English (63.4% de tweets). Valproic acid, instead, was not clearly mentioned by a predominant type of user; rather, it was more distributed among the user type studied. Undetermined users were more frequent in Spanish tweets than in English tweets, especially those discussing quetiapine (47.7%).

### Medical content results

**Fig. 2:**
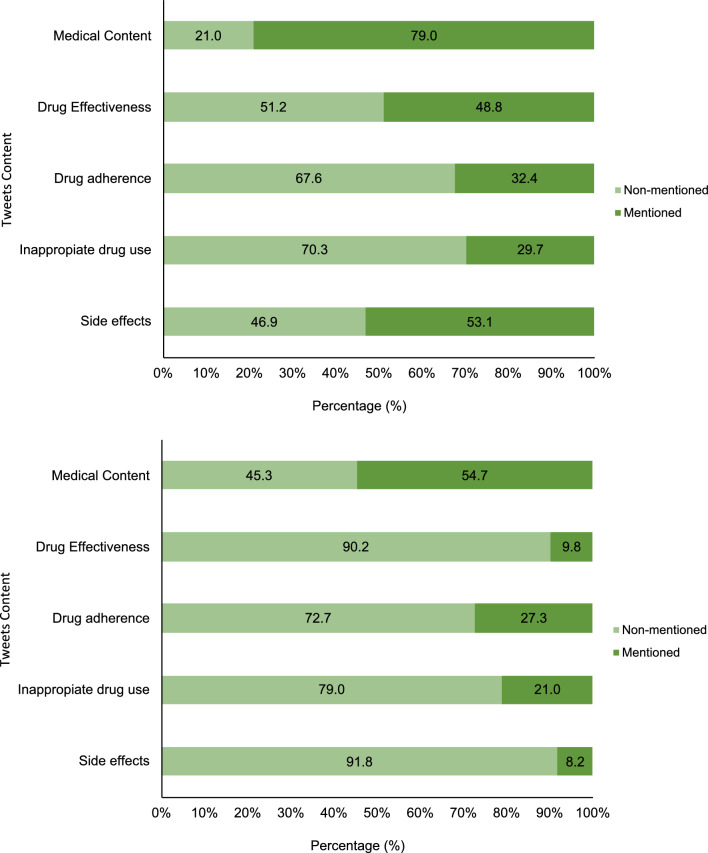
Proportion of tweets posted in English (top panel) and Spanish (bottom panel) of lithium, quetiapine and valproic acid, according to the clinical medical topics of the study. Clinical content consisted of drug effectiveness, categorising tweets if they talked about the effectiveness or not. Drug adherence categorised tweets as good adherence reported or did not mention adherence. Inappropriate use categorised tweets if they mentioned inappropriate use or did not mention inappropriate use. Understanding inappropriate use if they mentioned the use of the drug in a recreational way or clearly showing a different use rather than therapeutic. Side effects, classified tweets as the presence of side effects or did not mention side effects

As shown in Fig. 2 the overall proportion of tweets classified as enclosing medical content was higher in English tweets (79.0%) than in Spanish tweets (54.7%). Regarding specific medical themes, side effects were the most frequently mentioned topic in English tweets (53.1%), whereas in Spanish tweets, they only represented 8,2% of the tweets. Moreover, in Spanish tweets, the most frequently mentioned clinical topic was drug adherence (27.3%), with similar proportion (32.4%) as in English tweets. Proportion of tweets discussing inappropriate use content were similar in both languages (29.7% in English and 21.0% in Spanish).

### Other type of content analysis

**Fig. 3:**
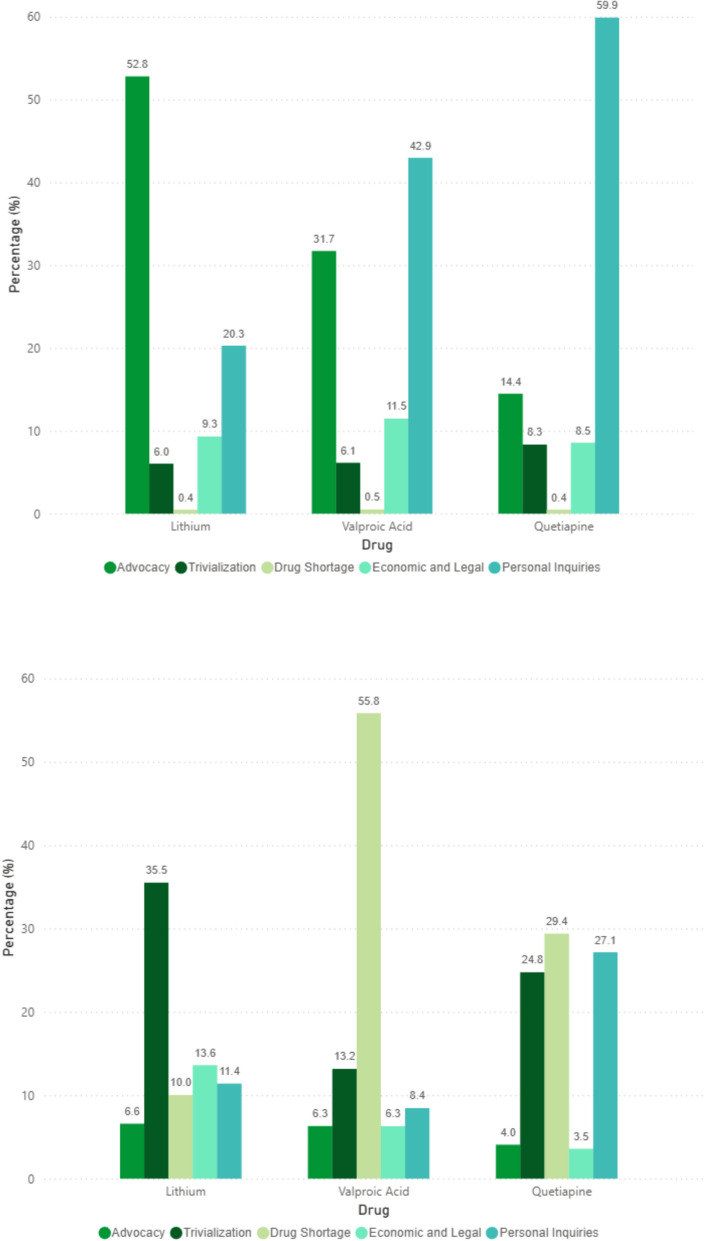
Grouped bar charts showing the distribution of other medical content across study medications in tweets posted in English (upper panel) and tweets posted in Spanish (lower panel). The Other type of content category classified tweets into the following subcategories: Personal inquiries, tweets in which users expressed doubts, concerns, or sought advice about these medications; Economic and legal activities, tweets referring to financial barriers, insurance coverage, price concerns, or regulatory issues related to these drugs; Advocacy, tweets promoting evidence-based information about these medications, raising awareness of benefits/risks, or sharing scientific knowledge; Drug shortage, tweets explicitly reporting difficulties accessing or finding the medication due to supply issues; and Trivialisation, tweets using these medications in jokes, memes, or sarcastic remarks, often mocking their effects or reputation

As shown in Fig. 3 there were notable differences in the distribution of other type of content across medications and languages. Among English tweets, lithium showed the highest proportion of tweets related to scientific advocacy (52.8%). Quetiapine had the highest percentage of personal inquiries (59.9%), and valproic acid was the drug most frequently associated with economic and legal concerns (11.5%). Mentions of drug shortages and trivialisation were relatively low across all three medications.

Regarding Spanish tweets, valproic acid stood out due to a markedly higher proportion of tweets reporting drug shortages (55.8%). Quetiapine and lithium were more frequently associated with trivialisation (24.8% and 35.5%, respectively), and quetiapine also concentrated a substantial number of personal inquiries (27.1%). Mentions of advocacy were visibly low for all three medications.

## Discussion

This study provides a comprehensive examination of how lithium, quetiapine, and valproic acid have been represented on X from 2008 to 2022. The findings of the study revealed substantial differences in thematic content, levels of engagement, and types of users across tweets posted in English and Spanish. Moreover, quetiapine was the most frequently mentioned medication; however, lithium generated significantly higher engagement.

Our results align with recent evidence highlighting a growing gap between clinical guidelines and real-world prescribing practices. For example, Singh et al. (2023) published a study involving 10,351 participants across North America, Europe, and Australia, assessing global prescription patterns for BD. The authors concluded that prescribing patterns did not align with major clinical guidelines, revealing notable differences across geographic regions. For instance, there was a higher prescription rate of second-generation antipsychotics and a lower prescription rate of lithium in North America compared to Europe (Singh et al. 2023). These results have also been described in many other regions worldwide (Adiukwu et al. 2025), pinpointing an important concern (Malhi et al. 2020), as lithium is still considered the gold standard treatment for BD in the main clinical guidelines (Yatham et al. 2018; Malhi et al. 2015). These patterns were mirrored in our dataset, where tweets authored by patients predominantly focused on quetiapine, whereas healthcare professionals and academic institutions more often discussed lithium. While social media activity cannot be equated directly with prescribing data, our findings provide complementary insights into the attitudes and perceptions that may influence clinical practice. Previous studies have demonstrated correlations between online discourse and real-world outcomes across different domains. For instance, alcohol-related tweets have been linked to regional consumption rates (Curtis et al. 2018), while social media activity has also been associated with patterns of opioid-related mortality (Sarker et al. 2019) and suicide rates (Wang et al. 2023). These findings suggest that online platforms may capture signals relevant to real-world behaviours. Nevertheless, further research is needed to confirm the consistency and reliability of this complementarity in the context of psychiatric medications.

Our analysis revealed that tweets discussing lithium were not only the most highly engaged but also closely associated with scientific advocacy, particularly in English tweets. These findings may denote a focus of interest and concern within academic and professional communities. However, a high level of trivialisation was also observed in Spanish tweets, in which mocking or sarcastic references were relatively common. Such narratives may foster negative perceptions and reinforce stigma, potentially deterring patients from initiating or continuing lithium treatment. This is especially concerning considering that adherence to medication in BD is low (Coldham et al. 2002; García et al. 2016), and attitudes toward treatment are among the strongest predictors of adherence (García et al. 2016). Our findings support these observations and point out social media as a promising platform for promoting psychoeducation, especially given the high level of engagement regarding tweets talking about lithium, which could help address misperceptions and improve clinician and patient confidence in lithium use.

In addition to lithium, both quetiapine and valproic acid received tweets referencing inappropriate use, with users describing non-therapeutic or recreational consumption. These findings align with literature documenting the misuse of psychiatric medications (Desantis et al. 2008; Schifano et al. 2018; Klein et al. 2017) and highlight the influence of social narratives on public attitudes. This finding suggests careful consideration of the extent to which content related to medications shapes social attitudes and stigma, particularly among patients and their families who may come across such information on social media, which could inadvertently hinder adherence. As previously reported, attitude towards medication is even more crucial for adherence than side effects (Scott and Pope 2002). Furthermore, this result reflects the immense potential of social media to immediately detect such inappropriate use and implement measures as swiftly as possible (Lee et al. 2021).

Interestingly, the proportion of tweets explicitly referring to medication adherence closely mirrored global adherence rates reported in the scientific literature (Coldham et al. 2002; García et al. 2016). This observation highlights the potential of social media analysis to reflect real-world clinical patterns. Previous studies have shown that online conversations can indeed align with epidemiological data; for instance, social media activity related to alcohol and opioid use has been found to correlate with regional patterns of consumption and misuse (Curtis et al. 2018; Sarker et al. 2016). However, further research is needed to confirm the consistency and reliability of these findings across different contexts and populations. Since adherence plays a crucial role in treatment outcomes and is often shaped by individual beliefs and perceptions, increasing the visibility of this issue on platforms like X may be highly beneficial. A greater online presence could foster peer support, improve health literacy, and reduce stigma surrounding non-adherence. Moreover, with the growing implementation of personalised strategies to monitor lithium levels (Zorrilla et al. 2023), strategic dissemination through social media could further support adherence by enhancing awareness and accessibility of these emerging tools.

Another important finding was the prominent discussion over drug shortages of valproic acid among Spanish tweets. The issue of valproic acid availability has already been reported in several Spanish-speaking countries, including Spain and parts of Latin America (CIMA 2023). This valuable information acquired through social media networks is often not as immediate and effective through traditional pharmacovigilance mechanisms (Song et al. 2023), demonstrating the great potential of research in social media networks. The lack of supply is a preventable cause of non-adherence, and proper information about it should be assessed before prescribing a medication as a stabiliser like valproic acid, which is known to be maintained over a long time in BD. Previous studies have mentioned the potential use of social media as a new pharmacovigilance tool that could help, for example, manage the lack of different medications more quickly (Song et al. 2023).

The findings of this study should be interpreted considering several limitations. The analysis was limited to English and Spanish tweets, capturing only a portion of global perspectives and linguistic diversity. Although these languages are widely spoken and provide large, heterogeneous datasets, restricting the analysis in this way may exclude important cultural and linguistic nuances in how lithium, quetiapine, and valproic acid are perceived and discussed. Future studies should extend this approach to additional languages and regions to achieve a more comprehensive understanding of global attitudes toward these medications. In addition, quetiapine and valproic acid are widely prescribed beyond BD. Quetiapine is commonly used across various areas of psychiatry, including schizophrenia, major depressive disorder, anxiety disorders, and insomnia (Taylor et al. 2014). Valproic acid, in addition to its psychiatric uses, is also widely prescribed in neurology for the treatment of epilepsy and other seizure disorders (Tomson et al. 2016). This broad clinical application makes it challenging to determine whether tweets specifically refer to BD or other indications. Therefore, the discourse captured in our analysis should be seen as reflecting the wider social perception of these medications, not solely their use in BD. Additionally, Lamotrigine, although a first-line treatment for bipolar depression, was not included in our analysis because it is not indicated for mania. Future studies should explore the social perception of lamotrigine, considering its importance for the depressive phases of BD, and expand this type of analysis to second-line treatments, including other antipsychotics, to offer a more comprehensive view of the discourse surrounding pharmacological management of BD.

Furthermore, Social Media discourse may overrepresent negative experiences with pharmaceutical treatments (e.g., adverse effects, dissatisfaction), a phenomenon often reported in online settings (Sharma et al. 2020; Leonardo et al. 2020). Our categorisation focused on content types rather than sentiment polarity; therefore, the proportions observed should not be interpreted as a definitive balance of positive versus negative outcomes, nor as a direct reflection of actual prescribing practices. In addition, older adults and socioeconomically marginalised populations are often underrepresented on social media platforms such as X, limiting the generalisability of these findings.

Nonetheless, our study presents several strengths that contribute to the robustness of the findings. First, the analysis covers tweets from 2008 to 2022, providing a comprehensive view of trends and discussions over an extended period. Second, we classified the tweets by user type, allowing for a more nuanced understanding of who engages in these conversations, such as patients or healthcare professionals. Third, by analysing tweets in both English and Spanish, we were able to highlight important linguistic and cultural differences in the discussion of BD medications, offering insights into varying regional perspectives. Finally, the content analysis enabled us to delve deeper into the nature of the discussions, identifying key themes such as medical content and other important areas, such as advocacy or shortages of these medications, which are critical for understanding broader social dynamics surrounding BD treatments.

## Conclusion

This study highlights the dynamic role of social media in shaping and reflecting public and professional discourse on commonly used psychiatric medications for BD. Lithium, despite being the least mentioned, generated the highest engagement, particularly among healthcare professionals, while quetiapine was the most frequently discussed, especially by patients. This contrast mirrors current prescription patterns and aligns with the growing scientific concern about the underuse of lithium. Our findings underscore the potential of platforms like X not only for psychoeducation but also for the early detection of inappropriate use and issues such as drug shortages, offering valuable insights to inform clinical practice and public health strategies.

## Supplementary Information

Additional file 1.

## Data Availability

The data that support the findings of this study are available from Twitter Binder. Still, restrictions apply to the availability of these data, which were used under license for the current study, and so are not publicly available. Data are, however, available from the authors upon reasonable request and with permission of the corresponding author.
