# Supplementary material for: Analysing lithium, quetiapine and valproic acid on social media: an infodemiology study
Source: Int J Bipolar Disord. 2025 Oct 24;13:28. doi: 10.1186/s40345-025-00395-6 (PMC12552198; doi:10.1186/s40345-025-00395-6)

**Supplementary Material**

**S1. List of keywords used to obtain tweets related to lithium**, **quetiapine y valproic acid.**

These keywords include both generic names and authorized brand names of first and second-line treatments used in the maintenance therapy of BD, as approved by the Federal Drug Administration (FDA), Spanish drug Agency AEM, or both regulatory agencies:

Litio, Lithium, Plenur, Lithium Carbonate ER, Lithobid, Eskalith, Lithonate, Acido Valproico, Valproic acid, Depakine, Depakine Crono, Depakene, Depakote, Quetiapina, Quetiapine, Psicotric, Psicotric Retard, Rocoz, Seroquel, Seroquel Prolong, Atrolak, Atrolak Prolong, Qudix, Qudix Prolong, Quentiax, Quentiax Prolong, Rocoz, Rocoz Prolong.

**Table S1: Explanation of categories classification:**

| Category and explanation | Example |
| --- | --- |
| Patient: Tweets in which the author explicitly identified themselves as someone diagnosed with a psychiatric disorder or currently using lithium, quetiapine, or valproic acid, sharing personal experiences, struggles, or reflections related to treatment. | *I have Bipolar Disorder Type 1 Asperger's Syndrome and a mild Tourette's condition. I've been on a lot of meds for over 10 years but lithium carbonate was recently added and my brain will take a while to adjust to that.”* |
| Patient’s acquaintance: Tweets in which the author described the experiences of a relative, friend, or close contact receiving one of the medications, without claiming to be the patient themselves. | *For anyone curious about Lithium, & how messed up it is: My son was diagnosed bipolar at 11. They put him on lithium a while. He was a drone. When they ‘took him off’ they did just that. No tapering. He was 12 & started climbing our 2nd floor balcony, in winter to try and ‘fly’.”* |
| Healthcare professionals and institutions: Tweets written by individuals who explicitly self-identified as healthcare providers, usually discussing prescribing practices, clinical experiences, or medical observations about bipolar disorder treatments. When possible, we also cross-checked the user’s biography for references to professional roles such as ‘Dr,’ ‘nurse,’ ‘therapist,’ or similar identifiers to support the classification. | *“I highly recommend Lithium and or anticonvulsants to treat bipolar disorder. Make sure you tell your doctor to check kidney function, however, or it could lead to kidney failure.”* |
| Efficacy: Tweets describing perceived effectiveness based on personal or observed experiences with symptom improvement or treatment response. | *“I have Bipolar 1 and take Lithium. 750 MG, but the dosage has changed many times. Been on it for a decade. It saved my life. I wrote a whole book of odes to it, coming out in September.”* |
| Inappropriate use: Tweets describing non-therapeutic or recreational consumption of these medications, often in combination with alcohol or other substances, or used episodically without medical supervision. | *“I was already a tad drunk and then my seroquel kicked in and now I just keep falling over.”* |
| Side effects: Tweets detailing side effects to the medications, including tolerability issues, physical or emotional effects. | *“Thanks to taking lithium for my bipolar it's made my thyroid underactive. Feeling well lethargic today.”* |
| Personal inquiries: Tweets in which users expressed doubts, concerns, or sought advice about these medications. | *“Also anyone developed psoriasis after taking lithium? I’ve had this psoriasis-like rash off and on in the years since I started taking it which just showed up again after being off it for a few days and starting it again. I wish I didn’t have to take all these medications!”* |
| Economic and legal activities: Tweets referring to financial barriers, insurance coverage, price concerns, or regulatory issues related to these drugs | *“Please everyone clap for me I dealt with a health insurance issue today (currently do not have it), called the health insurance company, AND called my pharmacy to try to figure out how I’m going to refill my Seroquel (I’m out after today) and I have not cried once!!”* |
| Advocacy: Tweets promoting evidence-based information about these medications, raising awareness of benefits/risks, or sharing scientific knowledge. | *Really important work by our large group at … Read the paper: Lithium for Suicide-Related Outcome Prevention in Veterans With Major Depression or Bipolar Disorder.”* |
| Drug shortage: Tweets explicitly reporting difficulties accessing or finding the medication due to supply issues. | *“My mother struggling to get Depakote (bipolar meds). Local where prescription sent hasn’t had stock all week. Does any nurse doctor pharmacist have any in stock? Not sure how collection can work but will travel to collect! Plz retweet!”* |
| Trivialisation: Tweets using these medications in jokes, memes, or sarcastic remarks, often mocking their effects or reputation. | *“If you're feeling a little depressed, put a small amount of lithium in your coffee.”* |

**Figure S2:** Tweet analysis classification flowchart


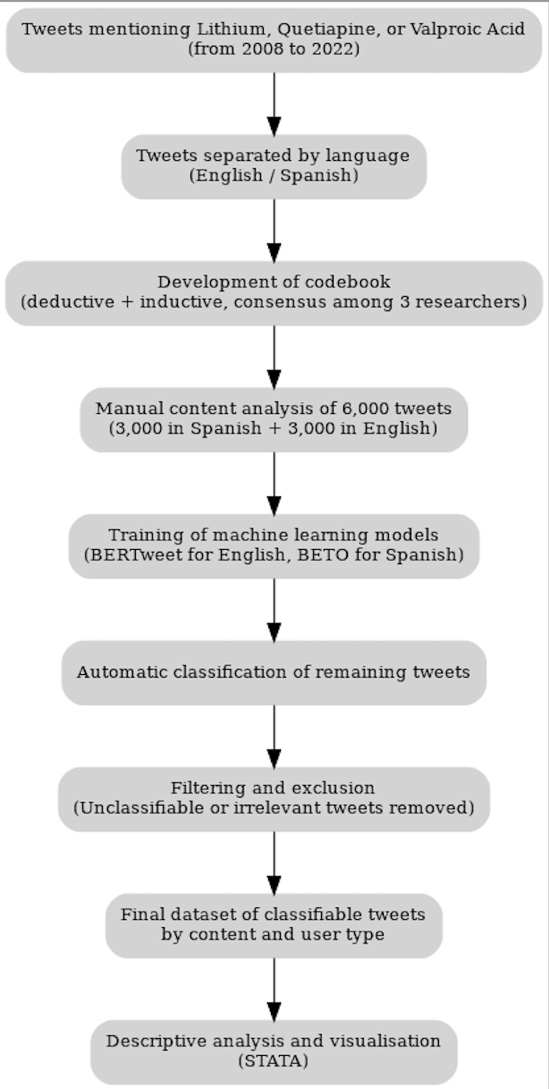

Supplement: Supplementary file 1 — Additional file 1. [file 40345_2025_395_MOESM1_ESM.docx]
